# Density Functional Study of Structures and Electron Affinities of BrO_4_F/BrO_4_F^−^

**DOI:** 10.3390/ijms10073128

**Published:** 2009-07-08

**Authors:** Liangfa Gong, Jieming Xiong, Xinmin Wu, Chuansong Qi, Wei Li, Wenli Guo

**Affiliations:** College of Chemical Engineering, Beijing Institute of Petro-Chemical Technology, Beijing 102617, China; E-Mails: gongliangfa@bipt.edu.cn (L.G.); wuxinmin@bipt.edu.cn (X.W.); qichuansong@bipt.edu.cn (C.Q.); liwei77@bipt.edu.cn (W.L.)

**Keywords:** density functional theory, bromine fluorine oxides, DFT-based descriptors, EA

## Abstract

The structures, electron affinities and bond dissociation energies of BrO_4_F/BrO_4_F^−^ species have been investigated with five density functional theory (DFT) methods with DZP++ basis sets. The planar F-Br…O_2_…O_2_ complexes possess ^3^A′ electronic state for neutral molecule and ^4^A′ state for the corresponding anion. Three types of the neutral-anion energy separations are the adiabatic electron affinity (EA_ad_), the vertical electron affinity (EA_vert_), and the vertical detachment energy (VDE). The EA_ad_ value predicted by B3LYP method is 4.52 eV. The bond dissociation energies D_e_ (BrO_4_F → BrO_4-m_F + O_m_) (m = 1–4) and D_e_^−^ (BrO_4_F^−^ → BrO_4-m_F^−^ + O_m_ and BrO_4_F^−^ → BrO_4-m_F + O_m_^−^) are predicted. The adiabatic electron affinities (EA_ad_) were predicted to be 4.52 eV for F-Br…O_2_…O_2_ (^3^A′←^4^A′) (B3LYP method).

## 1. Introduction

In recent days, density functional theory (DFT) has been enjoying tremendous success in electronic structure calculations for molecules and solids alike [1–8]. The DFT methods are able to describe the electronic structure of these systems with accuracies comparable to traditional correlated molecular orbital methods at a decreased computational cost. Furthermore these techniques are observed to assign more bonding character to the Lewis system in which the nucleophilic reaction occurs [9]. The DFT-based global and local properties (namely, DFT descriptors), such as Fukui functions, global and local hardness or softness [10,11], have already been used for reliable predictions in various types of electrophilic and nucleophilic reactions on a diversity of material structures [1–9,12–16]. In some sense, the DFT-descriptors provide us with more rigorous alternatives than the classical frontier orbital analysis. Chatterjee’s group have already used the DFT-descriptors for predictions in electrophilic and nucleophilic reactions in the case of zeolites and clay materials with or without solvent environment [1–7].

On the other hand, the bromine, chlorine, and fluorine oxides are known to be important in lower stratospheric ozone depletion, and have been the subjects of intense studies in recent years [17–26, and references cited therein]. Relevant bromine oxide fluorides, represent intriguing ternary molecules involving covalent bond between highly electronegative atoms, possessing a large number of unpaired electrons, resulting in strong lone pair-lone pair repulsions. Therefore, the hypervalent structures of these species could be characterized. As early as 1972, Johnson *et al*. [27] reported the thermodynamic properties of Br(VII) FBrO_3_ species. In 1976, Appelman *et al*. [28] characterized the molecular structure of gaseous perbromyl fluoride (FBrO_3_), and Gillespie and Spekkens [29] prepared and characterized potassium difluorodioxobromate (BrO_2_F_2_^−^) and tetrafluoro-oxobromate (BrOF_4_^−^). In 1978, Christe *et al*. [30] reported the vibrational frequencies and assignment of BrOF_3_. In 2005, Lehmann *et al*. [31] reported synthesis and characterization of salts containing the bromine (VII) BrO_3_F_2_^−^ anion; last year, Lehmann *et al*. [32] also reported the characterization of BrO_3_F and ClO_3_F to [XO_2_][SbF_6_] (X = Cl, Br) by single crystal X-ray diffraction, raman spectroscopy, and computational methods. The results showed that of a few computational methods, the DFT functional, B3LYP in combination with the aug-cc-pVTZ basis set, and the QCISD and CCSD(T) calculations provided the most reliable correlation with the experimental geometry and vibrational frequencies of BrO_2_^+^ [33] and likely provide reliable estimates of the geometric parameters and vibrational frequencies of BrO_3_^+^, as well as benchmarks for calculations involving bromine fluoride and oxide fluoride species [33]. Correspondingly, the density functional theory (DFT) in conjunction with DZP++ basis set has also localized these Br-hypervalent ternary structures to be minimum on the potential energy surfaces (PES) [34,35]. The planar/lineaer FBrO/FBrO-, pseudo-trigonal bipyramid F(F_2_)Br=O (C_s_ symmetric) [34] and [F-(:BrO_2_)-F]^−^ (C_2v_) anionic [29], and quasi-octahedral (OBr-F_4_)^−^ (C_4v_) [34,29] Br(V) structures have been found to be the lowest-lying isomers. However, the hypervalent FBrO_2_, FBrO_3_ [35], and their corresponding anionic isomers are local minima on the PES. These DFT methods, especially the hybrid DFT methods (BHLYP and B3LYP) are reliable to predict the bond lengths and bond angles [32]. Besides the rich fluoride chemistry of the III and V oxidation states of Br oxides, the fluoride ion transfer reactions containing Br(VII) are scarce and have only been established by the syntheses of the ternary bromine oxide fluorides, BrO_3_F_2_^−^ [31]. In this work, we report the systemic theoretical investigation of the similar BrO_4_F/BrO_4_F^−^ species, which may be of importance in atmospheric chemistry.

DFT/DZP++ scheme has been shown to be successful in prediction of electron affinities (EAs) of many species, such as BrOF_n_/BrOF_n_^−^, FBrO_2_/FBrO_3_, Br_2_O_n_/Br_2_O_n_^−^, BrClF_n_/BrClF_n_ and SF_5_O_n_/SF_5_O_n_^−^ (n = 1–3) species [34–38]. These studies and others have demonstrated that the DFT/DZP++ methods can predict electron affinities (EAs) in a good accuracy [39]. In addition, these methods are reliable for the geometry optimization of the neutral radicals and their anion.

The aim of the present work is to apply five DFT methods to predict the electron affinities of ternary bromine oxide fluoride, BrO_4_F, as well as the equilibrium geometries, harmonic vibrational frequencies, and bond dissociation energies. Four forms of the electron affinities are calculated, evaluated as the neutral–anion energy separations in the following manners. The adiabatic electron affinities (EA_ad_) are determined by, EA_ad_ = E_(optimized neutral)_ – E_(optimized anion)_, zero-point corrected adiabatic electron affinities (EA_zero_) are determined by, EA_zero_ = E_(zero-point corrected neutral)_ – E_(zero-point corrected anion)_, the vertical electron affinities (EA_vert_) by, EA_vert_ = ***E***_(optimized neutral)_ – ***E***_(anion at optimized neutral geometry)_, and the vertical detachment energies (VDE) of the anion by, VDE = ***E***_(neutral at optimized anion geometry)_ – *E*_(optimized anion)_. The DFT descriptors, such as Fukui functions, global and local hardness or softness [10,11], also have been used for the reliable predictions in the stability of BrO_4_F isomers.

## 2. Theory

Just like Chatterjee *et al*. [1–5] rationalized the structure-property relationship in different clays and observed that the hydroxyl groups present in the clay structure play a crucial role in the catalytic activity. We have explored the role of O and F atoms on the structure and properties of different bromine oxygen fluoides [34,35].

The hard-soft acid-base (HSAB) principles categorize the interaction between acids and bases in terms of global softness. Pearson proposed the global HSAB principle [40]. The global hardness was the second derivative of energy with respect to the number of electrons at constant temperature and external potential, which includes the nuclear field. The nonchemical meaning of the word “hardness” is resistance to deformation or change.

The global softness is the inverse of this. Pearson also pointed out a principle of maximum hardness (PMH) [41], which stated that, for a constant external potential, the system with the maximum global hardness is the most stable.

DFT-based local properties, like Fukui functions and local softness [10], have already been used for reliable predictions of electrophilic and nucleophilic reactions [1–8]. Generally, compared to a gas-phase calculation, the solvent environment alters the charge distribution of a molecule. There is an increase in the dipole moment of molecules such as water and BrF, which enhances the intrinsic reactivity of polar molecules toward nucleophilic and electrophilic attack [15]. Our aim in the current work is to explore the role of O_n_ chain in the structure and bonding of BrO_4_F species. DFT-based local descriptors have been used for calculating the reactivity index within the helm of the HSAB principle [11–15]. It is used to determine the possible correlation between BrO_4_F isomers.

In density functional theory, hardness (*η*) [40] is defined as:

$$\eta =(1/2)(\partial^{2}E/\partial N^{2})v(r)=(1/2)(\partial \mu /\partial N{)}_{v}$$

where *E* is the total energy, *N* is the number of electrons of the chemical species, and *μ* is the chemical potential.

The global softness, S, is defined as the inverse of the global hardness, *η*:

$$S=\frac{1}{2\eta }=(\partial N/\partial \mu {)}_{v}$$

Using the finite difference approximation, *S* can be approximated as:

(1) S=1/(IE−EA)

where *IE* and *EA* are the first ionization energy and electron affinity of the molecule, respectively.

The Fukui function *f(r)* is defined by [10]:

(2) $$f(r)=[\delta \mu /\delta v(r){]}_{N}=[\partial \rho (r)/\partial N{]}_{v}$$

The function *f* is thus a local quantity, which has different values at different points in the species, *N* is the total number of electrons, *μ* is the chemical potential, and *v* is the potential acting on an electron due to all nuclei present. Since *ρ(r)* as a function of *N* has slope discontinuities, equation 1 provides the following three reaction indices [10]:

$$\begin{array}{c} f^{-}(r)=[\partial \rho (r)/\partial N{]}_{v}^{-}\;(\text{governing}\;\text{electrophilic}\;\text{attack})\\ f^{+}(r)=[\partial \rho (r)/\partial N{]}_{v}^{+}\;\;\;(\text{governing}\;\text{nucleophilic}\;\text{attack})\\ f^{0}(r)=(1/2)[f^{+}(r)+f^{-}(r)]\;(\text{for}\;\text{radical}\;\text{attack}) \end{array}$$

In a finite difference approximation, the condensed Fukui function [16] of an atom, say x, in a molecule with *N* electrons is defined as:

(3) $$\begin{array}{c} f_{x}^{+}=[q_{x}(N+1)-q_{x}(N)]\;(\text{for}\;\text{nucleophilic}\;\text{attack})\\ \;f_{x}^{-}=[q_{x}(N)-q_{x}(N-1)]\;(\text{for}\;\text{electrophilic}\;\text{attack})\\ \;f_{x}^{0}=[q_{x}(N+1)-q_{x}(N-1)]/2\;(\text{for}\;\text{radical}\;\text{attack}) \end{array}$$

where *q*_x_ is the electronic population of atom x in a molecule. The local softness *s(r)* can be defined as:

(4) $$s(r)=(\delta \rho (r)/\delta \mu {)}_{v}$$

Equation(3) can also be written as:

(5) $$s(r)=[\partial \rho (r)/\partial N{]}_{v}[\partial N/\partial \mu {]}_{v}=f(r)S$$

Thus, local softness contains the same information as the Fukui function *f(r)* plus additional information about the total molecular softness, which is related to the global reactivity with respect to a reaction partner, as stated in the HSAB principle. Atomic softness values can easily be calculated by using equation 4, namely:

(6) $$\begin{array}{l} \;s_{x}^{+}(r)=[q_{x}(N+1)-q_{x}(N)]S\\ s_{x}^{-}(r)=[q_{x}(N)-q_{x}(N-1)]S\\ s_{x}^{0}(r)=S[q_{x}(N+1)-q_{x}(N-1)]/2 \end{array}$$

## 3. Methodology

The five different DFT exchange-correlation functionals employed in this work range from generalized gradient approximation (GGA) [BLYP, BP86] to hybrid-GGA [BHLYP, B3P86, and B3LYP]. These hybrid Hartree-Fock/density functionals include: (a) Becke’s half and half HF/DFT hybrid exchange functional (BH) [42] with the Lee, Yang, and Parr correlation functional (LYP) [43] (BHLYP); (b) Becke’s three parameter functional [44] (B3) plus Perdew’s correlation functional (P86) [45] (B3P86); (c) B3 combined with LYP functionals (B3LYP) [44,43]; (d) incorporation of Becke’s 1988 exchange functional (B) [46] with Perdew’s correlation functional (P86) (BP86); (e) B along with LYP (BLYP) [46,43]. The standard double-*ζ* plus polarization (DZP) basis set augmented with diffuse functions (DZP++) were utilized. The basis set for bromine was comprised of Ahlrichs’ standard doublẹ-*spd* set plus a set of *d*-type polarization functions [*α_d_* (Br) = 0.389] [47] plus diffuse functions [*α_s_* (Br) = 0.0469 and *α_p_* (Br) = 0.0465]. For oxygen and fluorine, the basis sets were composed of the standard Huzinaga-Dunning [48,49] doublẹ-*ζ* set plus one set of polarization functions [*α_d_* (O) = 0.85, *α_d_* (F) = 1.00] augmented with one set of diffuse functions [*α_s_* (O) = 0.08227, *α_p_* (O) = 0.06508, and *α_s_* (F) = 0.1049, *α_p_* (F) = 0.0826]. The final contracted basis sets are thus designated as Br (15s12p6d/9s7p3d), O (10s6p1d/5s3p1d), and F (10s6p1d/5s3p1d). All of the molecular structures and the electron affinities have been determined using the Gaussian 03 program suite [50]. The fine integration grid (99 590) was used. All stationary point geometries were characterized by the evaluation of their harmonic vibrational frequencies at the five different levels of theory. Unless otherwise reported, the geometries in figures were found to be minima after determining the harmonic vibrational frequencies via analytical second derivatives for the corresponding stationary point structures for each function.

Besides the electron affinities, the bond dissociation energies for BrO_4_F/BrO_4_F^−^ are also determined as the difference in total energies in the following manners:

The bond dissociation energies for the neutrals refer to the reactions:

$${\text{BrO}}_{4}\text{F}\to {\text{BrO}}_{3}\text{F}+\text{O},\;{\text{BrO}}_{4}\text{F}\to {\text{BrO}}_{2}\text{F}+{\text{O}}_{2},\;{\text{BrO}}_{4}\text{F}\to \text{BrOF}+{\text{O}}_{3},$$

The bond dissociation energies for the anions refer to the reactions:

$${\text{BrO}}_{4}{\text{F}}^{-}\to {\text{BrO}}_{3}{\text{F}}^{-}+\text{O},\;{\text{BrO}}_{4}{\text{F}}^{-}\to {\text{BrO}}_{2}{\text{F}}^{-}+{\text{O}}_{2},\;{\text{BrO}}_{4}{\text{F}}^{-}\to {\text{BrOF}}^{-}+{\text{O}}_{3},$$

$${\text{BrO}}_{4}{\text{F}}^{-}\to {\text{BrO}}_{3}\text{F}+{\text{O}}^{-},\;{\text{BrO}}_{4}{\text{F}}^{-}\to {\text{BrO}}_{2}\text{F}+{{\text{O}}_{2}}^{-},\;{\text{BrO}}_{4}{\text{F}}^{-}\to \text{BrOF}+{{\text{O}}_{3}}^{-}$$

The natrural bond orbital (NBO) analysis [51] was carried out at the B3LYP/DZP++ level for some species, corresponding Wiberg bond index (WBI) and atomic charges are obtained. Unless otherwise stated, we use the B3LYP result for molecular structures and energetics. The counterpoise (CP) method [52] was used to correct the basis set superposition error (BSSE) [7,53] using the Boys-Bernardi method in the calculation of the binding energy for the current basis. For these complexes of Lewis species, the single point calculations of the cation and anion of each molecule at the optimized geometry of the neutral molecule were also carried out to evaluate Fukui functions, global and local softness [10]. The condensed Fukui function and atomic softness were evaluated using equations 3 and 6 in Section 2. Theory, respectively. The gross atomic charges were evaluated using the technique of Mulliken charges, due to the Br atomic charge can hardly be evaluated by using the technique of electrostatic potential (ESP) driven charges.

## 4. Results and Discussion

With the present five DFT methods, the optimized O-F bond length for single OF molecule ranges from 1.331 Å (BHLYP) to 1.385 Å (BLYP) (not shown). The trend of bond lengths predicted for O-F is BHLYP < B3P86 < B3LYP < BP86 < BLYP. The DZP++ B3LYP method gives the result closest to the experimental O-F bond length (r_e_) of 1.3541 Å, obtained from Raman spectroscopy [18 and references cited therein]. The B3LYP method also obtain the best prediction result for dissociation energy (D_e_) of OF [23] and BrO [21]. For a discussion of the reliability of B3LYP thermochemistry, see the recent work of Boese, Martin, and Handy [54]. Therefore, in the following discussion, unless otherwise stated, we use the B3LYP result for molecular structures and energetics.

For neutral BrO_4_F species, the molecular chain FBr…OO…OO structure with a terminal F-Br moiety connected by OO…OO chain lies the lowest energetically. This structure in its ^5^A′ state (all of the five DFT methods) or ^3^A′ state (both BP86 and BLYP pure DFT methods) corresponds a very loose van der Waals complex between BrF…OO and O_2_, possessing a binding energy of about zero and the very long Br…O (2.719–3.004 Å in ^5^A′ state and 2.618, 2.719 Å in ^3^A′ state) and (O)O^…^O(O) (5.220–7.095 Å in ^5^A′ state, and 5.746, 6.014 Å in ^3^A′ state) distances (not shown). It is favorable to dissociate into BrF + 2O_2_ (^3^Σ_g_^−^) or BrF + O_2_ (^1^Δ_g_) + O_2_ (^3^Σ_g_^−^).

**Figure 1.**
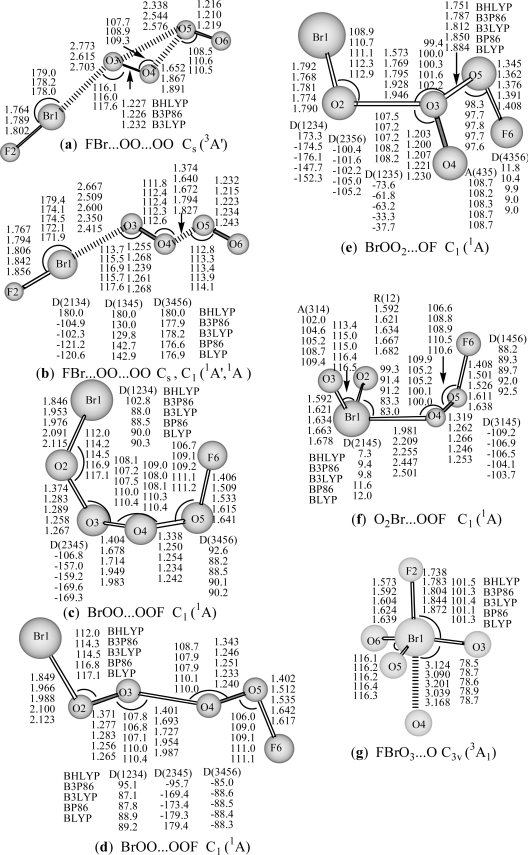
Optimized geometries of neutral BrO_4_F (a-g) with DFT/DZP++ approach (bond lengths in Å, bond angles and dihedral angles in degrees). A: represents bond angle, D: represents torsion angle.

**Figure 2.**
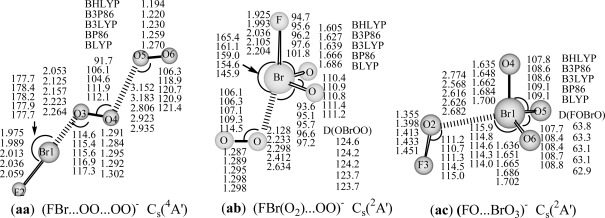
Optimized geometries of anionic BrO_4_F^−^ **(aa-ac)** with DFT/DZP++ approach (bond lengths in Å, bond angles and dihedral angles in degrees). A: represents bond angle, D: represents torsion angle.

The FBr…OO…OO structures in ^3^A′ state (**a:** ^3^A′) optimized by three hybrid DFT methods (BHLYP, B3P86 and B3LYP) and in ^1^A′ (**b:** ^1^A′) (BHLYP) or ^1^A (**b:** ^1^A) state (with the rest four DFT methods) are reported in Figure 1. The optimized geometries for both Br- and F-terminal structures, including cis- and trans- BrOO…OOF (**c:** ^1^A and **d:** ^1^A), and BrOO(O)…OF (**e:** **^1^**A), and those of Br-hypervalent structures, O_2_Br…OOF (**f:** **^1^**A) and FBrO_3_...O (**g:** C_3v_, ^3^A_1_) are also displayed in Figure 1. The optimized geometries for anionic BrO_4_F^−^ species, including (FBr…OO)^−^…OO (**aa**: ^4^A′) chain, and [FBr(O_2_)...OO]^−^ (**ab**: ^2^A′), (FO…BrO_3_)^−^ (**ac**: ^2^A′) Br-hypervalent structures are shown in Figure 2. They may represent an important intermediate in atmospheric reactions.

The calculated energies (Table 1) show that the FBr…OO…OO structure in its ^5^A′ state or its dissociation products (FBr...OO (^3^A″) + O_2_ (^3^Σ_g_^−^)) lies lower than the corresponding ^3^A′ (**a**) and ^1^A′ or ^1^A (**b**) states by about 33 and 60 kcal/mol respectively with the B3LYP method. This state also lies much lower than the cis-, trans- BrOO…OOF (**c:** ^1^A and **d:** ^1^A) and BrOO_2_…OF (**e:** **^1^**A) isomers by ca.64, 64, and 95 kcal/mol (Table 1) respectively (B3LYP). The O_2_Br…OOF (**f:** **^1^**A) and FBrO_3_...O (**g:** C_3v_, ^3^A_1_) Br-hypervalent structures lie much higher than the ^5^A′ state by ca. 78 and 130 kcal/mol (Table 1) respectively. With a few exceptions, the two pure DFT methods (BP86 and BLYP) predict much smaller relative energies and the bond dissociation energies than three hybrid DFT methods. All these discrepancies indicate that BrO_4_F is a challenging target for DFT methods.

As can be seen from Figure 1, for the FBr…OO…OO structure in its ^3^A′ state, the covalent bond lengths are predicted to be 1.764–1.802 Å for the Br-F bond, and 1.227–1.232 Å for interim O-O and 1.216–1.219 Å for the terminal O-O bond, and the complex bond distances are 2.615–2.773 Å for Br…O, and 1.652–1.891 Å for (O)O^…^O(O) with three hybrid DFT methods. At the B3LYP level, Br-F bond length, the interim O-O and terminal O-O bond lengths in the ^3^A′ state (**a** in Figure 1) are 1.802, 1.232, and 1.219 Å respectively, and Br…O or (O)O^…^O(O) complex distance is 2.703 or 1.891 Å respectively. These structure parameters are similar to those of the corresponding ^1^A′ state or ^1^A state of (**b** in Figure 1), in which, the Br-F bond distance, the interim O-O and terminal O-O bond lengths (**b** in Figure 1) are slightly elongated (1.806, 1.239, and 1.223 Å respectively at B3LYP level), and Br…O or (O)O^…^O(O) complex distance is significantly shortened (2.600 or 1.672 Å respectively). The geometric and electronic structures show that the F-Br terminal moiety connected by OO…OO chain structure in ^3^A′ (hybrid DFT methods) or in singlet state may be viewed as a van der Waals complex between BrF moiety and OO-OO covalent-like chain respectively. NBO analyses (B3LYP) show that the ^3^A′ state possesses stronger single Br-F (WBI: 0.795 vs 0.781) and double O-O (WBI: 1.462 vs 1.436 for interim O-O; 1.524 vs 1.519 for terminal O-O) bonds than the singlet state, and that the covalent OO-OO (WBI: 0.418 vs 0.717) and complex Br…O (WBI: 0.063 vs 0.089) bonds in ^3^A′ state are weaker than those in singlet state. Compared with the ^3^A″ state of FBr...OO [35], the Br-F and interim O-O bonds in ^3^A′ state of FBr^...^OO-OO are slightly elongated by 0.01 Å, whereas Br^...^O distance is significantly shorter by 0.26 Å (B3LYP), and the terminal O-O bond distance is very similar to that in free O_2_ (^3^Σ_g_^−^) (1.194–1.245 Å) [55].

It is worthy to note that the geometries predicted using the five functionals are all similar, with small variations in bond lengths and angles. The general trend for the covalent bond lengths is BLYP > BP86 > B3LYP > B3P86 > BHLYP. According to previous studies on geometries of BrOF_n_/BrOF_n_^−^, FBrO_2_/FBrO_3_, BrClF_n_/BrClF_n_ and BrF_n_ species [34,35,37,56], the hybrid DFT methods (BHLYP, B3P86 or B3LYP method) are excellent methods for the prediction of covalent bond lengths. The B3LYP method taking the median position may be regarded as a compromise between the reliabilities of geometry and thermochemical parameter predictions. This order coincides with that predicted for the FO molecule [25] where comparison with experiment indicates the B3LYP method to be the most accurate in prediction of geometry, and for BrO in predictions of bond dissociation and adiabatic electron affinity (EA_ad_) [21].

The attachment of an electron to FBr…OO…OO complex, results in the ^4^A′ ground state for anion (**aa**: ^4^A′ in Figure 2). As might be expected, this structure is more stable than other anionic BrO_4_F^−^ Br-hypervalent structures (**ab**: ^2^A′ and **ac**: ^2^A′ in Figure 2) by 32 and 78 kcal/mol at B3LYP/DZP++ level. The covalent bond lengths are predicted to be 1.975–2.059 Å for the Br-F bond, and 1.284–1.302 Å for interim O-O and 1.194–1.270 Å for the terminal O-O bond, and the complex bond distances are 2.053–2.264 Å for Br…O, and 2.806–3.183 Å for (O)O^…^O(O) in the ^4^A′ state of BrO_4_F^−^. Comparison with the similar neutral isomer shows that there is a substantial change in geometry between neutral ^3^A′ state and anionic ^4^A′ state. The Br-F bond (2.013 Å at B3LYP level), the interim O-O bond (1.295 Å) and Br…O bond (2.157 Å) in anionic ^4^A′ state are analogous to those of (FBr-OO)^−^ (2.038 Å for Br-F, 1.302 Å for O-O, and 2.135 Å for Br…O) [35]; the terminal O-O bond in the ^4^A′ state of BrO_4_F^−^ (1.230 Å) is similar to that of free O_2_ (1.219 Å at B3LYP level) [55]; the (O)O^…^O(O) distance of 2.806 Å in anionic BrO_4_F^−^ is substantially longer than the corresponding (O)O^…^O(O) distance (1.891 Å) in ^3^A′ state of neutral BrO_4_F. Thus, this BrO_4_F^−^ structure in ^4^A′ state could be regarded as a van der Waals complex between (FBr-OO)^−^ [35] and O_2_ (^3^Σ_g_^−^) due to suitable Br…O and (O)O…O(O) bonding distances, and the high negative charge of FBr-OO moiety (near to −1 from NBO analysis). Neither theoretical nor experimental values of BrO_4_F/BrO_4_F^−^ are available for comparison. For this structure in its doublet ^2^A′ state, the results of all five DFT methods are suspect due to the large spin contamination, with <S^2^> = 1.77 or 1.76.

For the cis- and trans- BrOO…OOF (**c:** ^1^A and **d:** ^1^A in Figure 1) structures, the bond lengths are calculated to be 1.846–2.123 Å for the Br-O bond (that in cis-form tinily shorter than in trans-like), 1.402–1.642 Å for the F-O bond, 1.401–1.987 Å for the central single O–O bond, and 1.233–1.374 Å for outer O–O bonds connected by Br and F. In the cis- BrOO…OOF, the O…OO fragment nearly in a planar, both BrO and FO bonds are almost perpendicular to this planar, however, in the trans- BrOO…OOF, the OO…OO chain nearly in a planar, the BrO and FO bonds are also almost perpendicular to this planar. At B3LYP level, both BrOO…OOF isomers nearly possess the same stability. This BrOO…OOF conformation could be viewed as a complex comprising of unstable BrOO and FOO molecules, furthermore, the DFT methods predict it thermodynamic instability with respect to dissociation into BrOO + FOO (not shown).

For the BrOO_2_…OF (**e:** **^1^**A) structures, the bond lengths are calculated to be 1.768–1.792 Å for the Br-O bond, 1.345–1.408 Å for the F-O bond, 1.200–1.230 Å for the central double O–O bond and 1.573–1.946 Å, 1.7514–1.884 Å for outer single O–O bonds connected by Br and F, respectively. At B3LYP level, the Br-O (1.781 Å) or F-O (1.376 Å) bond is slightly longer than that in BrO [21] or FO [25]. Thus, this BrOO_2_…OF (**e:** **^1^**A) structures could be regarded as a complex comprising of simple BrO, O_2_ and FO molecules. The hybrid DFT methods predict it thermodynamic instability with respect to dissociation into BrO + O_2_ + OF (not shown), whereas the pure DFT methods predict the reaction energy of about 10 (BP86) and 6 kcal/mol (BLYP) for BrOO_2_…OF (**e:** **^1^**A) → BrO + O_2_ + OF (not shown).

For Br-hypervalent structures: O_2_Br…OOF (**f:** **^1^**A), the bond lengths are predicted to be 1.592–1.682 Å for Br-O_term_ (with an oxygen atom at the terminal position), 1.981–2.501 Å for Br-O_mid_ (with O atom at the middle position), and 1.246–1.319 Å for O-O, and 1.408–1.638 Å for F-O. The predicted Br-O_term_ length is comparable to that of OBrO (1.649 Å) [21], F-O or O-O bond distances are slightly shorter or longer than those in FOO (1.649 or 1.200 Å) [18]. This structure could be thought as a complex between BrO_2_ and FOO. Likewise, the hybrid DFT methods predict it thermodynamic instability with respect to dissociation into BrO_2_ + FOO (not shown), and the pure DFT methods predict the dissociation energy of O_2_Br…OOF (**f:** **^1^**A) → BrO_2_ + FOO reaction is about 2.3 (BP86) and 0.5 kcal/mol (BLYP) respectively (not shown).

For the rare Br(VII) FBrO_3_...O (**g:** C_3v_, ^3^A_1_) complex between FBrO_3_ and O atom, the bond lengths are predicted to be 1.573–1.639 Å for Br-O, 1.738–1.832 Å for Br-F, and 3.039–3.168 Å for Br…O. At B3LYP level, Br-O bond long is 1.604 Å, analogous to that in BrO_4_^−^ (1.603 Å) [21] or BrO_3_F_2_^−^ (1.601 Å) [31], and longer than that in FBrO_3_ (1.582 Å) [35], however, significantly shorter than that in BrO_3_^−^ (1.648 Å) [21]. The Br-F bond length is 1.804 Å, being significantly shorter than that in BrO_3_F_2_^−^ (1.872 or 1.849 Å) [31], while substantially longer than that in FBrO_3_ (1.708 Å) [35]. ∠FBrO and ∠OBrO angles are 101.4 and 116.2° respectively, slightly narrower than those in FBrO_3_ theoretically (101.9 and 115.9) or experimentally (103.3 and 114.9°) [27]. Generally, the predicted lengths are comparable to those of FBrO_3_ (C_3v_) and BrO_3_F_2_^−^ anion [31]. The DFT methods predict the dissociation energy of FBrO_3_...O (**g:** C_3v_, ^3^A_1_) → FBrO_3_ (C_3v_) + O reaction is about 1 kcal/mol (Table 1), demonstrating that this Br(VII) FBrO_3_...O (**g:** C_3v_, ^3^A_1_) hypervalent structure is bound for dissociation to FBrO_3_ and O.

The corresponding anion eventually to dissociation into FBr(O_2_)^−^...OO (**ab**: ^2^A′) complex structure, Br-F and Br-O bonds are elongated to be 2.036 and 1.639 Å (B3LYP), the Br…O complex distance and O-O bond length are about 2.3 and 1.30 Å. The DFT methods predict the dissociation energy of FBr(O_2_)^−^...OO (**ab**: ^2^A′) → BrF^−^ + O_2_ (^3^Σ_g_^−^) +O_2_ (^1^Δ_g_) reaction being in the range of 7–48 kcal/mol, the BHLYP result is too small (7 kcal/mol). This is not unexpected, given the large fraction of exact exchange in the BHLYP method [57]. For the global minimum FBr…OO…OO anion (**aa**: ^4^A′), the predictions of five different DFT methods for the dissociation energies for **aa** to dissociate to its components [FBr...OO^−^(^2^A″) + O_2_, FBr...OO (^3^A″) + O_2_^−^, or BrF^−^+ 2O_2_(^3^Σ_g_^−^)] show the same trend, i.e. the pure DFT (BP86 and BLYP) methods predict higher dissociation energies than the hybrid DFT methods, and the BHLYP result is the smallest.

For the higher-lying hypervalent anionic (FO…BrO_3_)^−^ complex structure (**ac**: ^2^A′), the bond lengths are predicted to be 1.635–1.702 Å for Br-O bonds, 1.355–1.451Å for F-O bond, 1.355–1.451Å for F-O bond, and 2.568–2.774 Å for Br…O complex bond. The theoretical dissociation energies for (FO…BrO_3_)^−^ → BrO_3_^−^ (C_3v_) + FO is in the range of 2.8–17.9 kcal/mol (Table 2). Likewise, the pure DFT methods predict higher dissociation energies, and the BHLYP result is the lowest.

Generally, the theoretical dissociation energies (D_e_) for BrO_4_F/BrO_4_F^−^ species can be evaluated from the data in Tables 1 and 2. For the anionic BrO_4_F^−^ species, all of five DFT methods predict almost consistent relative energies and bond dissociation energies, with the exception of the lowest BHLYP results (Table 2) (vide supra). In contrast, for the neutral BrO_4_F species (Table 1), the relative energies and bond dissociation energies predicted by BHLYP method are nearly the biggest. It is noted that BHLYP method perform poorly for bond-breaking process [57] due to the large (50%) contribution from Hartree-Fock or exact exchange. Based on the previous studies of the BrO_n_ species [21] and the anionic BrO_4_F^−^ species (vide supra), the B3LYP methods should predict reasonable dissociation energies and relative energies, however, caution is urged because of the complex of BrO_4_F ternary system.

At B3LYP level, for the lowest energies species, the theoretical bond dissociation energies for neutral BrO_4_F refer to the reactions: BrO_4_F→ BrO_4-m_F + O_m_ (m = 1–4). For BrO_4_F → BrO_2_F (^3^A″) [35] + O_2_, the theoretical reaction energies (ca. zero) are much smaller than those of BrO_4_F→ BrO_3_F (^1^A’) + O (range from 84 to 109 kcal/mol, about 100 kcal/mol at B3LYP level) and BrO_4_F→ BrOF (^1^A’) + O_3_ (range from 48 to 101 kcal/mol, ca. 71 kcal/mol at B3LYP level), indicating the dissociation reaction is favored, which is consistent with the FBr…O_2_…O_2_ complex structure.

The most reliable B3LYP method predicts the dissociation energy (D_e_) for F-Br…O_2_…O_2_ (^5^A′) → BrF + 2O_2_ and (F-Br…O_2_…O_2_)^−^ (^4^A′) → BrF^−^ + 2O_2_ are only 0.0 and 9.1 kcal/mol, respectively (Tables 1 and 2), suggesting a weak van der Waals interaction between the BrF or BrF^−^ and O_2_ moieties.

For the anionic BrO_4_F^−^ species, the D_e_ of BrO_4_F^−^ → BrO_4-m_F^−^ + O_m_ and BrO_4_F^−^ → BrO_4-m_F + O_m_^−^predicted (Table 2). The bond dissociation energies for BrO_4_F^−^ → BrO_2_F^−^ + O_2_ are smaller positive values, from 1.0 to 1.5 kcal/mol for three hybrid DFT methods and 4.4 or 4.9 kcal/mol for BP86 or BLYP (two pure DFT) methods. The D_e_ values predicted by BHLYP method are too low to be reliable. The D_e_ value of 1.4 kcal/mol predicted by B3LYP is much smaller than those of BrO_4_F^−^ → BrO_3_F^−^ + O (71 kcal/mol) and BrO_4_F^−^ → OBrF^−^ + O_3_ (60 kcal/mol).

For BrO_4_F^−^ → BrO_4-m_F + O_m_^−^ reactions, the higher bond dissociation energies are predicted, the D_e_ value (58 kcal/mol) of BrO_4_F^−^ → BrO_2_F + O_2_^−^ is also smaller than those of BrO_4_F^−^ → BrO_3_F + O^−^ (136 kcal/mol) and BrO_4_F^−^ → OBrF + O_3_^−^ (81 kcal/mol), and demonstrating that complex BrO_n_F [34,35] species have higher electron affinities than the free O_m_ species [55]. For the challenging BrO_m_F/BrO_m_F^−^ (m = 1–4) species, minima on PES were found with all of DFT methods employed. However, the thermodynamic stabilities decrease with n (vide supra).

The EA_ad_ for FBr-O_2_-O_2_ (a: ^3^A′**← aa**: ^4^A′) are predicted to be 4.95 eV(BHLYP), 4.97 eV(B3P86), and 4.52 eV(B3LYP), zero-point corrected EA_ad_ (EA_zero_) is only increased about 0.05 eV. At B3LYP level, EA_zero_ is 4.57 eV, larger than those of FBr-OOO [35] and FBrO [34] by about 0.1 and 1.9 eV respectively, and much smaller than those of FBr-OO by 1.3 eV (B3LYP). Those with odd n (n = 1 and 3, closed shell) have smaller EAs than those of species for the even number of n (n = 2 and 4), which are open-shell triplet state. The EA_vert_ values range from 2.13 to 3.55 eV. The range of VDE is from 4.49 to 4.98eV. No experimental data are available.

The harmonic vibrational frequencies and IR active intensities of BrO_4_F/BrO_4_F^−^ species predicted by B3LYP method are available in Tables 3 and 4. For triplet state FBr...O_2_...O_2_ (**a**) (*C_s_*, ^3^A′), the calculated infrared spectrum is characterized by three strong bands around 1561 (terminal O-O symmetri stretch(s.s.)), 1440 (middle O-O s.s.), 628 cm^−1^(F-Br s.s.), all other modes give rise to weak intensities. For singlet state FBr...OOOO (b) (C_1_, ^1^A), the bands of ca. 1508 (terminal O-O s.s.), 1391 (middle O-O s.s.), and 620 cm^−1^(F-Br s.s.) possess the stronger intensities. For BrOO...OOF chain structures (**c**) and (**d**), the predicted infrared spectrum are characterized by three stronger bands around 1223, 1376 (F-connected O-O s.s.), 1107, 1276 (Br-connected O-O s.s.), and 720, 667 cm^−1^(F-O-O bend), respectively, the rest modes yield weak intensities. For BrOO2...OF structure (e), four bands around 1209, 934, 862, and 718 cm^−1^ exist the stronger intensities, the corresponding modes are O-O (O2) s.s., F-O s.s., Br-O s.s., and O...O stretch.

For O_2_Br...OOF structure (**f**), four bands around 1529, 1055, 732, and 618 cm^−1^ possess the stronger intensities, the corresponding modes are O-O stretch, OBrO asymmetric bend, OBrO symmetric bend, and FOO bend. For the highest symmetric Br(VII) FBrO_3_...O (**g**), theoretical infrared spectrum are characterized by the stronger bands around θ: 955 cm^−1^ (BrO_3_ asymmetric stretch (a.s.)); η: 864 cm^−1^ (BrO_3_ symm.bend); ζ: 567 cm^−1^(F-Br s.s.); ɛ: 364 cm^−1^(OBrO in the planar bend); δ: 345 cm^−1^ (OBrO out of planar bend), the harmonic vibrational frequencies of BrO_3_ radical are larger than the corresponding BrO_3_^+^ [32] (966, 850, 329, and 231 cm^−1^). For anionic quartet state FBr...OO...OO (**aa**) (C_s_, ^4^A′) species, four bands around 1532, 1226, 383, and 227 cm^−1^ possess the stronger intensities. For anionic hypervalency structure [FO...Br(O)O_2_] ^−^ (**ac**) (C_s_, ^2^A′), four bands around 957, 812, 805, 789 cm^−1^ possess the stronger intensities.

Isodesmic reactions, which have been typically used to obtain the heats of formation for many molecules, are those in which the reactants and products contain the same types of bonds, i.e., the number of bonds broken and formed is conserved [58]. An isodesmic reaction scheme requires that the heats of formation of all the molecules involved in the reaction to be known with the exception of the heat of formation of the particular isomer. Because of this property, errors in the energy that might occur due to defects in the basis set and electron correlation cancel, to a large extent. The isodesmic scheme used here is BrOOOOF + 4HOH → 3HOOH + HOBr + HOF. During the calculation of the heat of formation of BrOOOOF using the isodesmic scheme, literature values for the heats of formation of HOH (−57.10 kcal mol^−1^) [59], HOOH (−31.02 kcal mol^−1^) [59], and HOBr (−10.93 kcal mol^−1^) [60], HOF (−22.47 kcal mol^−1^) [61], were used. Using these results we were able to calculate the heats of reaction. For cis BrOOOOF (**c**), the heat of formation is predicted to be 50 kcalmol^−1^ at B3LYP level of theory (Table 5). Using the relative energies (Table 1) along with the heat of formation of BrOOOOF (**c**), we obtained a value of 19 kcal mol^−1^ for FBrOOOO(**a**), 83 kcal mol^−1^ for BrOO2…OF (**e**), 64 kcal mol^−1^ for O_2_Br…OOF (**f**), and 116 kcal mol^−1^ for FBrO_3_…O (**g**) (shown in Table 6). To further assess these results, we have listed all five DFT methods heats of formation of the isomers in Table 6. At present, there are no experimental measurements to which be mainly due to the incompleteness of the basis sets and only partial allowance for electron correlation.

For these complexes of Lewis acid (BrF) and base (lone pair O_m_ chains), we treated as a local version of the hard and soft acid base (HSAB) principle [40]. The DFT-based local reactivity descriptors such as the global or local softness or hardness, condensed Fukui functions can be used to explain the stability of isomers. The predicted global hardness (*η*) and softness (*GS*) for the minimum-energy BrO_4_F structures (**a**, **b**, **c**, **d**, **e**, **f**, and **g** isomers) with five DFT methods are shown in Tables 7 and 8 respectively. The local softness (S_x_^+^ and S_x_^−^), and ratios of them (S_x_^−^/S_x_^+^) for the minimum BrO_4_F structures (**a**, **b**, **c**, **d**, **e**, **f**, and **g** isomers) at the B3LYP/DZP++ level are tabulated in Table 9. According the Pearson’s PMH suggestion [41], the Br(VII) structure (**g**) FBrO_3_…O in this work has the largest global hardness (Table 7), and smallest global softness (Table 8), thus triplet state FBrO_3_…O structure is the most stable isomer. For BrO_4_F isomers, the maximum value (from 5.1 to 8.2, at B3LYP/DZZ++ level, as 8.2) of global hardness (Table 7) set in the highest symmetric Br(VII) FBrO_3_...O structure (**g**), whereas the minimum value (from 2.9 to 3.2) of hardness assign to singlet BrOO...OOF isomer (**b**), inversely, the isomers (**g**) or (**b**) possesses the smallest or largest global softness (Table 8), respectively, namely, from 0.06 to 1.0, or from 0.16 to 0.17. For Br in the different isomers presents almost either the largest or smallest S_x_^−^/S_x_^+^ values (Table 9), corresponding to different bonds stabilities. An important finding from this investigation is that Br may reveal the flexibility in which the bromine atom shares valence electrons and orbitals to form a variety of hypervalent species, even the extend hypervalent system.

## 5. Conclusions

The structures, electron affinities and bond dissociation energies of BrO_4_F/BrO_4_F^−^ species have been studied with five DFT methods. The B3LYP method is the most reliable method for predicting the geometry and electron affinities for this ternary species. The EA_ad_ value predicted by the B3LYP method is 4.52 eV for BrO_4_F. The EA_ad_ values for OBrF [34], FBrOO, and FBrOOO [35] species are 3.64, 5.83 and 4.43 eV, respectively. and close to those of other interhalogen compounds, such as BrCIF_n_ and BrF_n_ [37,56]. Those with odd n (n = 1 and 3, closed shell) have smaller EA_ad_ than those of even n (n = 2 and 4) species, which are open-shell triplet state. These substantial electron affinities suggest that the corresponding anion may have the lifetimes as independent species under atmospheric conditions.

Similar to the case of the electron affinities, the hybrid DFT methods especial BHLYP predict the discrepant values of bond dissociation energies for BrO_4_F/BrO_4_F dissociation reactions and relative energies from two pure DFT methods, demonstrating that this system is a challenge for DFT methods.

Although the FBr-O_2_-O_2_/(FBr-O_2_-O_2_)^−^ chain structures have been found to be the most stable isomers, yet there is no workable reaction mechanism for the formation of these species considering only BrF or BrF^−^, BrO and O_2_ or O_2_^−^ as starting materials. According recently report on bromine (VII) BrO_3_F_2_^−^ anion [31], we conclude that the Br(VII) structure, FBrO_3_...O (**g:** C_3v_, ^3^A_1_) are the most likely structure for neutral BrO_4_F, and the BrO_4_F^−^ may have Br(V) (FO…BrO_3_)^−^ (**ac**: ^2^A′) complex structure. The DFT-based local reactivity descriptors such as the global or local softness or hardness, condensed Fukui functions can demonstrate this suggestion.

The DFT methods are able to describe the electronic structure of these systems with accuracies comparable to traditional correlated molecular orbital methods at a decreased computational cost. Furthermore these DFT-based local descriptors techniques are observed to assign more bonding character to the BrO_4_F Lewis system.

## Figures and Tables

**Table 1.** Relative energies in kcal·mol^−1^ for BrO_4_F and its dissociation products species a

	**BHLYP**	**B3P86**	**B3LYP**	**BP86**	**BLYP**
**FBr...OO...OO(^5^A**″**)**	0.00	0.00	0.00	0.00	0.00
**FBr...OO (^3^A″) + O_2_ (^3^Σ_g_^−^)^c^**	0.00	0.00	0.00	0.00	0.00

**(a) (C_s_,^3^A′)**	56.08	31.27	33.03	---b	---b
**(b) (C_s_, ^1^A′)**	106.42	57.41	60.41	21.72	24.59
**(c) (C_1_, ^1^A)**	88.48	60.12	64.18	31.04	34.06
**(d) (C_1_, ^1^A)**	87.86	60.49	64.22	31.31	34.97
**(e) (C_1_, ^1^A)**	136.87	91.65	94.63	60.82	62.94
**(f) (C_1_, ^1^A)**	112.37	72.28	78.33	43.66	48.31
**(g) (C_3v_, ^3^A_1_)**	134.71	123.75	129.94	118.89	124.35
**FBr^...^OOO (^1^A′) + O^c^**	109.33	100.48	99.94	86.41	84.45
**FBr^...^OO (^3^A″) + O_2_(^1^Δ_g_)^c^**	43.19	39.65	39.15	38.02	38.90
**OBrF (^1^A′) + O_3_^c^**	100.63	67.12	70.73	47.95	51.31

^a^ corrected with ZPVE.

^b^ At pure DFT methods (BP86 and BLYP), the triplet state of F-Br…O_2_…O_2_ dissociated to BrF and O_2_.

^c^ The bond dissociation energies corrected with BSSE.

^d^ BrOO is not converge with hybrid DFT methods.

**Table 2.** Relative energies (corrected with ZPVE) in kcal mol^−1^ for the BrO_4_F^−^ species a

	**BHLYP**	**B3P86**	**B3LYP**	**BP86**	**BLYP**
**aa (C_s_, ^4^A′)**	0.00	0.00	0.00	0.00	0.00
**ab(C_s_, ^2^A′)**	44.67	27.77	32.06	22.02	25.84
**ac (C_s_, ^2^A′)**	89.41	74.36	78.47	69.67	73.64
**FBr...OOO^−^ (^2^A″)+ O b**	62.73	72.17	71.24	78.52	77.23
**FBr...OO^−^ (^2^A″) + O_2_^b^**	1.02	1.49	1.38	4.42	4.90
**OBrF^−^+ O_3_^b^**	72.04	59.70	60.46	52.44	54.10
**FBr...OOO(^1^A′) + O^−^b**	143.20	137.42	135.97	127.36	126.87
**FBr...OO (^3^A″) + O_2_^−^b**	50.96	60.66	57.87	69.20	67.16
**OBrF (^1^A′) + O_3_^−^b**	92.18	79.32	80.61	75.51	76.23
**BrF^−^+ 2O_2_(^3^Σg^−^)^b^**	−1.39	11.42	9.06	20.79	19.59
**BrO_3_^−^ + OF b**	92.59	86.40	90.39	87.94	92.27

^a^ corrected with ZPVE.

^b^ The bond dissociation energies corrected with BSSE.

**Table 3.** Predicted total energies(E_total_) in hartree, zero-point vibrational energies (ZPE) in kcal mol^−1^, and harmonic vibrational frequencies (Freq) in cm^−1^and the infrared intensities (in parenthese, in km·mol^−1^) for the minimum-energy BrO_4_F (**a**, **b**, **c**, **d**, and **e**) structures at the B3LYP/DZP++ level.

**Isomers**	**a (C_s_, ^3^A′)**	**b (C_1_, ^1^A)**	**c (C_1_, ^1^A)**	**d (C_1_, ^1^A)**	**e (C_1_, ^1^A)**
**E_total_**	−2974.61676	−2974.57337	−2974.56896	−2974.56840	−2974.54599
**ZPE**	7.37	7.88	8.53	8.22	8.05
**Freq**	ω_1_(a″) 27 (<1)	ω_1_ 13 (<1)	ω_1_ 28 (<1)	ω_1_24 (<1)	ω_1_ 79 (<1)
	ω_2_(a′) 35 (1)	ω_2_ 61 (1)	ω_2_ 97 (1)	ω_2_ 59 (1)	ω_2_ 98 (<1)
	ω_3_(a″) 67 (<1)	ω_3_ 81 (3)	ω_3_ 175 (3)	ω_3_ 103 (<1)	ω_3_ 133 (0)
	ω_4_(a′) 68 (5)	ω_4_ 97 (7)	ω_4_ 249 (7)	ω_4_ 201 (0)	ω_4_ 211 (14)
	ω_5_(a″) 96 (1)	ω_5_ 110 (2)	ω_5_ 287 (3)	ω_5_ 252 (4)	ω_5_ 247(2)
	ω_6_(a′) 102 (2)	ω_6_ 225 (1)	ω_6_ 367(13)	ω_6_ 281 (36)	ω_6_ 338 (16)
	ω_7_(a′) 206 (4)	ω_7_ 292 (5)	ω_7_ 501 (44)	ω_7_ 444 (28)	ω_7_ 451 (3)
	ω_8_(a′) 276 (10)	ω_8_ 345 (3)	ω_8_ 546(33)	ω_8_ 496 (63)	ω_8_ 502 (32)
	ω_9_(a′) 628 (132)	ω_9_ 620 (142)	ω_9_ 664 (60)	ω_9_ 571 (1)	ω_9_ 718 (148)
	ω_10_(a′) 650 (<1)	ω_10_ 766 (<1)	ω_10_ 720 (74)	ω_10_ 667 (172)	ω_10_862(86)
	ω_11_(a′)1440(643)	ω_11_1393 (529)	ω_11_ 1107 (105)	ω_11_ 1276 162)	ω_11_ 934(93)
	ω_12_(a′)1561(110)	ω_12_1508 (278)	ω_12_ 1223 (112)	ω_12_ 1376 (69)	ω_12_1209(161)

**Table 4.** Predicted total energies(E_total_) in hartree, zero-point vibrational energies (ZPE) in kcal mol^−1^, and harmonic vibrational frequencies (Freq) in cm^−1^and the infrared intensities (in parenthese, in km mol^−1^) for the minimum-energy BrO_4_F/BrO_4_F^−^ (**f**, **g/aa**, **ac**) structures at the B3LYP/DZP++ level.

**Isomers**	**f (C_1_, ^1^A)**	**g (C_3v_, ^3^A_1_)**	**aa (C_s_, ^4^A′)**	**ac (C_s_,^2^A′)**
**E_total_**	−2974.52034	−2974.46217	−2974.78297	−2974.65675
**ZPE**	8.47	7.28	6.22	7.03
**Freq**	ω_1_ 41 (<1)	ω_1_(e) α 39 (<1)	ω_1_(a′) 13 (<1)	ω_1_(a″) 31 (1)
	ω_2_ 104 (0)	ω_2_(a_1_)β 66 (<1)	ω_2_(a″) 16 (<1)	ω_2_(a′) 56 (3)
	ω_3_ 110(1)	ω_3_(e) γ 269 (<1)	ω_3_(a″) 34 (<1)	ω_3_(a″) 77 (4)
	ω_4_ 231 (4)	ω_4_(a_1_)δ 345 (25)	ω_4_(a′) 49 (3)	ω_4_(a′) 90 (2)
	ω_5_ 246(5)	ω_5_(e) ɛ 364 (30)	ω_5_(a′) 102 (5)	ω_5_(a′) 200 (8)
	ω_6_ 283 (4)	ω_6_(a_1_)ζ 567 (144)	ω_6_(a′) 151 (29)	ω_6_(a″) 327 (14)
	ω_7_ 438 (30)	ω_7_(a_1_)η 864 (21)	ω_7_(a″) 181 (7)	ω_7_(a′) 327 (14)
	ω_8_ 535 (5)	ω_8_(e) θ 955 (106)	ω_8_(a′) 227 (45)	ω_8_(a′) 390 (63)
	ω_9_ 618 (6)		ω_9_(a′) 383 (636)	ω_9_(a′) 789 (100)
	ω_10_732 (42)		ω_10_(a′) 437 (25)	ω_10_(a′) 805 (174)
	ω_11_1055 (131)		ω_11_(a′)1226 (116)	ω_11_(a′) 812 (181)
	ω_12_1529 (360)		ω_12_(a′)1532 (1291)	ω_12_(a′) 957 (397)

**Table 5.** Isodesmic heats of reaction (kcal mol^−1^) and heats of formation of BrOOOOF (**c**)

**Levels**	**Total energies (hartrees)**					**Δ*H*^0^_r,0_ BrOOOOF + 4H_2_O → 3H_2_O_2_ + HOBr + HOF**	**Δ*H*^0^_f,0_ (BrOOOOF)**
	**HOH**	**HOBr**	**HOOH**	**HOF**	**BrOOOOFc (C_1_, ^1^A)**		
**BHLYP**	−76.40988	−2649.83736	−151.51249	−175.50871	−2974.29785	38.66	63.28
**B3P86**	−76.62947	−2650.88786	−151.91207	−175.90318	−2976.08610	52.80	49.14
**B3LYP**	−76.45274	−2649.92233	−151.59656	−175.59224	−2974.56896	51.98	49.96
**BP86**	−76.45287	−2650.17532	−151.60450	−175.59915	−2974.89229	76.51	24.74
**BLYP**	−76.43467	−2649.91747	−151.57898	−175.58312	−2974.61332	75.55	25.70

**Table 6.** Heats of formation (kcal mol^−1^) of BrO_4_F isomers.

	**a**	**b**	**c**	**d**	**e**	**f**	**g**
**BHLYP**	30.88	81.19	63.28	62.65	63.28	62.65	109.51
**B3P86**	20.30	46.44	49.14	49.51	83.45	61.30	112.77
**B3LYP**	18.81	46.49	49.96	50.00	82.94	64.11	115.72
**BP86**	---a	15.41	24.74	25.01	56.76	37.36	112.59
**BLYP**	---a	16.23	25.70	25.71	56.54	39.95	116.00

^a^ At pure DFT methods (BP86 and BLYP), the triplet state of F-Br…O_2_…O_2_ dissociated to BrF and O_2_.

**Table 7.** Global hardness Approximated as: *η* = 1/2(*IE* − *EA*) of BrO_4_F isomers.

	**a**	**b**	**c**	**d**	**e**	**f**	**g**
**BHLYP**	4.7887	3.0850	4.9960	4.9734	4.1040	4.6173	6.0183
**B3P86**	4.1283	3.1846	4.3096	4.2192	3.7400	4.0823	5.0613
**B3LYP**	4.1415	2.8950	4.1995	4.1144	3.5957	3.9306	8.2104
**BP86**	---a	3.1694	4.8052	3.6550	3.5028	3.8778	6.9996
**BLYP**	---a	3.1108	3.6132	3.5698	3.4107	3.7634	7.0271

^b^ At pure DFT methods (BP86 and BLYP), the triplet state of F-Br…O_2_…O_2_ dissociated to BrF and O_2_.

**Table 8.** Global softness approximated as: *GS* = 1/(2*η*) = 1/(*IE* − *EA*) of BrO_4_F isomers.

	**a**	**b**	**c**	**d**	**e**	**f**	**g**
**BHLYP**	0.1044	0.1621	0.1001	0.1005	0.1218	0.1083	0.0831
**B3P86**	0.1211	0.1570	0.1160	0.1185	0.1337	0.1225	0.0988
**B3LYP**	0.1207	0.1727	0.1191	0.1215	0.1391	0.1272	0.0609
**BP86**	---a	0.1578	0.1041	0.1368	0.1427	0.1290	0.0714
**BLYP**	---a	0.1607	0.1384	0.1401	0.1466	0.1329	0.0712

^b^ At pure DFT methods (BP86 and BLYP), the triplet state of F-Br…O_2_…O_2_ dissociated to BrF and O_2_.

**Table 9.** Predicted global softness (GS), local softness (S_x_^+^ and S_x_^−^), and ratio of them for the BrO_4_F (**a**, **b**, **c**, **d**, **e**, **f**, and **g**) isomers.

**Species**	**GS**	**atom**	**S_x_^+^**	**S_x_^−^**	**S_x_^0^**	**S_x_^−^/S_x_^+^**
**a**	0.1207	Br1	0.0139	0.0524	0.0332	3.7739
		F2	0.0091	0.0173	0.0132	1.8959
		O3	0.0321	0.0097	0.0209	0.3024
		O4	0.0191	0.0096	0.0144	0.5014
		O5	0.0136	0.0114	0.0125	0.8398
		O6	0.0329	0.0203	0.0266	0.6171
**b**	0.1727	Br1	0.0329	0.0695	0.0516	2.1350
		F2	0.0152	0.0248	0.0202	1.6460
		O3	0.0391	0.0240	0.0317	0.6206
		O4	0.0303	0.0089	0.0196	0.2964
		O5	0.0188	0.0077	0.0133	0.4142
		O6	0.0363	0.0359	0.0363	0.9989
**c**	0.1191	Br1	0.0758	0.0529	0.0643	0.6976
		O2	0.0025	0.0143	0.0084	5.6573
		O3	0.0120	0.0102	0.0111	0.8584
		O4	0.0067	0.0100	0.0083	1.5076
		O5	0.0122	0.0178	0.0150	1.4537
		F6	0.0100	0.0139	0.0119	1.4003
**d**	0.1215	Br1	0.0771	0.0540	0.0656	0.7007
		O2	0.0021	0.0144	0.0083	6.7342
		O3	0.0116	0.0102	0.0109	0.8789
		O4	0.0064	0.0094	0.0079	1.4708
		O5	0.0113	0.0165	0.0139	1.4627
		F6	0.0131	0.0170	0.0150	1.3029
**e**	0.1391	Br1	0.0451	0.0634	0.0542	1.4053
		O2	0.0212	0.0243	0.0228	1.1459
		O3	0.0057	0.0027	0.0042	0.4809
		O4	0.0294	0.0225	0.0260	0.7664
		O5	0.0243	0.0137	0.0190	0.5635
		F6	0.0133	0.0124	0.0128	0.9313
**f**	0.1272	Br1	0.0343	0.0174	0.0259	0.5084
		O2	0.0242	0.0234	0.0238	0.9666
		O3	0.0256	0.0341	0.0298	1.3307
		O4	0.0164	0.0168	0.0166	1.0268
		O5	0.0122	0.0179	0.0150	1.4651
		F6	0.0146	0.0176	0.0161	1.2120
**g**	0.0609	Br1	0.0094	−0.0046	0.0024	−0.4827
		F2	0.0072	0.0069	0.0070	0.9580
		O3	0.0065	−0.0008	0.0029	−0.1171
		O4	0.0249	0.0608	0.0429	2.4447

## References

[1] Chatterjee A Iwasaki T Hayashi H Ebina T Torri K Electronic and structural properties of montmorillonite—a quantum chemical study J. Mol. Catal 1998 136 195 202

[2] Chatterjee A Ebina T Mizukami F Effects of Water on the structure and bonding of resorcinol in the interlayer of montmorillonite nanocomposite: A periodic first principle study J. Phys. Chem. B 2005 109 7306 7313 16851836 10.1021/jp045775z

[3] Chatterjee A Iwasaki T Ebina T Miyamoto A A DFT study on clay–cation–water interaction in montmorillonite and beidellite Comput. Mater. Sci 1999 14 119 124

[4] Chatterjee A Iwasaki T Ebina T A novel method to correlate layer charge and the catalytic activity of 2:1 dioctahedral smectite clays in terms of binding the interlayer cation surrounded by monohydrate J. Phys. Chem A 2000 104 8216 8223

[5] Chatterjee A Ebina T Onodera Y Mizukami F Effect of exchangeable cation on the swelling property of 2:1 dioctahedral smectite—A periodic first principle study J. Chem. Phys 2004 120 3414 3422 15268498 10.1063/1.1640333

[6] Chatterjee A Iwasaki T Ebina T Sorbent for dioxins and furans: reactivity index study J. Phys. Chem. A 2002 106 641 648

[7] Chatterjee A Niwa S Mizukami F Structure and property correlation for Ag deposition on α-Al_2_O_3_ - a first principle study J. Mol. Graph. Model 2005 23 447 456 15781187 10.1016/j.jmgm.2005.01.002

[8] Szöllösi G Chatterjee A Forgó P Bartók M Mizukami F Structure-property relationship in py-hexahydrocinchonidine diastereomers: ab Initio and NMR study J. Phys. Chem. A 2005 109 860 868 16838957 10.1021/jp045882t

[9] Parr RG How I came about working in conceptual DFT Chemical Reactivity Theory: A Density Functional Theory View Chattaraj PK Taylor & Francis Group London, UK 2009

[10] Parr RG Yang W Density functional approach to the frontier-electron theory of chemical reactivity J. Am. Chem. Soc 1984 106 4049 4050

[11] Geerlings P De Proft F Langenaekar W Conceptual density functional theory J. Chem. Rev 2003 103 1793 1873 10.1021/cr990029p 12744694

[12] Nguyen LT Le TN De Proft F Chandra AK Langenaeker W Nguyen MT Geerlings P Mechanism of [2 + 1] cycloadditions of hydrogen isocyanide to alkynes: molecular orbital and density functional theory study J. Am. Chem. Soc 1999 121 5992 6001

[13] Langenaeker W De Proft F Geerlings P Ab initio and density functional theory study of the geometry and reactivity of benzyne, 3-fluorobenzyne, 4-fluorobenzyne, and 4,5-Didehydro-pyrimidine J. Phys. Chem. A 1998 102 5944 5950

[14] Chandra AK Geerlings P Nguyen MT On the asynchronism of isocyanide addition to dipolarophiles: Application of local softness J. Org. Chem 1997 62 6419

[15] Sivanesan D Amutha R Subramanian V Nair BU Ramaswami T Assessment of the importance of the solvent in the calculation of condensed Fukui function: a self-consistent reaction field calculation study Chem. Phys. Lett 1999 308 223 228

[16] Yang W Mortier MJ The use of global and local molecular parameters for the analysis of the gas-phase basicity of amines J. Am. Chem. Soc 1986 108 5708 5711 10.1021/ja00279a008 22175316

[17] Hebestreit K Stutz J Rosen D Matveiv V Peleg M Luria M Platt U DOAS Measurements of tropospheric bromine oxide in mid-latitudes Science 1999 283 55 57 9872738 10.1126/science.283.5398.55

[18] Karton A Parthiban S Martin JML Post-CCSD(T) ab initio thermochemistry of halogen Oxides and related hydrides XOX, XOOX, HOX, XO, and HXO (X = F, Cl), and evaluation of DFT methods for these systems J. Phys. Chem. A 2009 113 4802 4816 19159205 10.1021/jp8087435

[19] Matus MH Nguyen MT Dixon DA Peterson KA Francisco JS ClClO_2_ is the most stable isomer of Cl_2_O_2_. Accurate coupled cluster energetics and electronic spectra of Cl_2_O_2_ isomers J. Phys. Chem. A 2009 112 9623 9627 18778040 10.1021/jp806220r

[20] Li Z Francisco JS A density functional study of structure and heat of formation for Br_2_O_4_ and Br_2_O_5_ Chem. Phys. Lett 2002 354 109 119

[21] Xie Y Schaefer HF Wang Y Fu X Liu R Electron affinities of the bromine oxides BrO_n_, n = 1–4 Mol. Phys 2000 98 879 890

[22] Martin JML Heats of formation of perchloric acid, HClO4, and perchloric anhydride, Cl_2_O_7_. Probing the limits of W1 and W2 theory J. Mol. Struct.: THEOCHEM 2006 771 19 26

[23] Ju XH Wang ZY Yan XF Xiao HM Density functional theory studies on dioxygen difluoride and other fluorine/oxygen binary compounds: Availability and shortcoming J. Mol. Struct.: THEOCHEM 2007 804 95 100

[24] Prascher BP Wilson AK A computational study of dihalogen-l-dichalcogenides: XAAX (X = F, Cl, Br; A = S, Se) J. Mol. Struct. : THEOCHEM 2007 814 1 10

[25] Feller D Dixon DA Coupled cluster theory and multireference configuration interaction study of FO, F_2_O, FO_2_, and FOOF J. Phys. Chem. A 2003 107 9641 9651

[26] Kraka E He Y Cremer D Quantum chemical descriptions of FOOF: the unsolved problem of predicting its equilibrium geometry J. Phys. Chem. A 2001 105 3269 3276

[27] Johnson GK O’Hare PAG Appelman EH Thermodynamic properties of perbromyl fluoride (BrO_3_F) Inorg. Chem 1972 11 800 802

[28] Appelman EH Beagley B Cruickshank DWJ Foord A Rustad S Ulbrecht V An electron-diffraction study of the molecular structure of gaseous perbromyl fluoride and calculation of its force field and vibrational amplitudes J. Mol. Struct. : THEOCHEM 1976 35 139 148

[29] Gillespie RJ Spekkens P Preparation and characterization of potassiumdifluorodioxobromate and tetrafluoro-oxobromate J Chem Soc Dalton Trans 1976 2391 2396

[30] Christe KO Curtis EC Bougon R Bromine trifluoride oxide. Vibrational spectrum, force constants and thermodynamic properties Inorg. Chem 1978 17 1533 1539

[31] Lehmann JF Schrobilgen GJ Synthesis and characterization of salts containing the BrO_3_F_2_^−^ anion; A rare example of a bromine (VII) species J. Am. Chem. Soc 2005 127 9416 9427 15984869 10.1021/ja0402607

[32] Lehmann JF Riedel S Schrobilgen GJ Behavior of BrO_3_F and ClO_3_F Toward strong lewis acids and the characterization of [XO_2_][SbF_6_] (X = Cl, Br) by single cCrystal X-ray diffraction, raman spectroscopy, and computational methods Inorg. Chem 2008 47 8343 8356 18700751 10.1021/ic800929h

[33] Francisco JS Structure, vibrational spectra and energetics of OBrO^+^ Chem. Phys. Lett 1998 288 307 310

[34] Gong L Li Q Xie Y Schaefer HF Novel bromine oxyfluorides: Structures, thermochemistry and electron affinities of BrOF_n_/BrOF_n_^−^ (n = 1–5) Mol. Phys 2005 103 1995 2008

[35] Li S Gong L Wu X Guo W Structures and electron affinities of BrO_2_F and BrO_3_F Chem. Phys. Lett 2007 439 395 401

[36] Pak C Xie Y Schaefer HF Electron affinities of the dibromine oxides: Br_2_O_n_ (n = 0–4) Mol. Phys 2003 101 211 225

[37] Ignatyev IS Schaefer HF Bromine halides: The neutral molecules BrClF_n_ (n = 1–5) and their anions structures, energetics, and electron affinities J. Am. Chem. Soc 1999 121 6904 6910

[38] Xu W Cheng S Lu SX Structures, vibrational frequencies, and electron affinities of SF_5_O_n_/SF_5_O_n_^−^ (n = 1–3) J. Mol. Struct. : THEOCHEM 2009 900 77 83

[39] Rienstra-Kiracofe JC Tschumper GS Schaefer HF Nandi S Ellison GB Atomic and molecular electron affinities: Photoelectron experiments and theoretical computations Chem. Rev 2002 102 231 282 11782134 10.1021/cr990044u

[40] Parr RG Pearson RG Absolute Hardness: Companion parameter to absolute electronegativity J. Am. Chem. Soc 1983 105 7512 7516

[41] Pearson RG Recent advances in the concept of hard and soft acids and bases J. Chem. Educ 1987 64 561 567

[42] Becke AD A new mixing of Hartree–Fock and local density-functional theories J Chem Phys 1993 98 1372 BHandHLYP method in the Gaussian programs has 0.5*Ex(LSDA)+0.5*Ex(HF)+0.5*Delta-Ex(B88)+Ec(LYP) formula, which is *not* precisely the formulation proposed in his paper.

[43] Lee C Yang W Parr RG Development of the Colle-Salvetti correlation-energy formula into a functional of the electron density Phys. Rev. B 1988 37 785 789 10.1103/physrevb.37.785 9944570

[44] Becke AD Density-functional thermochemistry. III. The role of exact exchange J. Chem. Phys 1993 98 5648 5652

[45] Perdew JP Density-functional approximation for the correlation energy of the inhomogeneous electron gas Phys. Rev. B 1986 33 8822 8824 10.1103/physrevb.33.8822 9938299

[46] Becke AD Density-functional exchange-energy approximation with correct asymptotic behavior Phys. Rev. A 1988 38 3098 3100 9900728 10.1103/physreva.38.3098

[47] Schafer A Horn H Ahlrichs R Br Basis sets. Fully optimized contracted Gaussian basis sets for atoms Li to Kr J. Chem. Phys 1992 97 2571 2577

[48] Huzinaga S F and O Basis setsGaussian-Type Functions for Polyatomic Systems I J. Chem. Phys 1965 42 1293 1302

[49] Dunning TH Gaussian Basis Functions for Use in Molecular Calculations. I. Contraction of (9s5p) Atomic Basis Sets for the First-Row Atoms F and O Basis sets J. Chem. Phys 1970 53 2823 2833

[50] Frisch MJ Trucks GW Schlegel HB Scuseria GE Robb MA Cheeseman JR Jr Montgomery JA Vreven T Kudin KN Burant JC Millam JM Iyengar SS Tomasi J Barone V Mennucci B Cossi M Scalmani G Rega N Petersson GA Nakatsuji H Hada M Ehara M Toyota K Fukuda R Hasegawa J Ishida M Nakajima T Honda Y Kitao O Nakai H Klene M Li X Knox JE Hratchian HP Cross JB Adamo C Jaramillo J Gomperts R Stratmann RE Yazyev O Austin AJ Cammi R Pomelli C Ochterski JW Ayala PY Morokuma K Voth GA Salvador P Dannenberg JJ Zakrzewski VG Dapprich S Daniels AD Strain MC Farkas O Malick DK Rabuck AD Raghavachari K Foresman JB Ortiz JV Cui Q Baboul AG Clifford S Cioslowski J Stefanov BB Liu G Liashenko A Piskorz P Komaromi I Martin RL Fox DJ Keith T Al-Laham MA Peng CY Nanayakkara A Challacombe M Gill PMW Johnson B Chen W Wong MW Gonzalez C Pople JA Gaussian 03: IA32W-G03RevC 02 12-Jun-2004 Gaussian, Inc Wallingford, CT, USA 2004

[51] Reed AE Curtiss LA Weinhold F Intermolecular interactions from a natural bond orbital, donor-acceptor viewpoint Chem. Rev 1988 88 899 926

[52] Boys SF Bernardi F The calculation of small molecular interactions by the differences of separate total energies. Some procedures with reduced errors Mol. Phys 1970 19 553 557

[53] Orita H Itoh N Inada Y A comparison of CO adsorption on Pt(211), Ni(211), and Pd(211) surfaces using density functional theory Surf. Sci 2004 571 161 172

[54] Boese AD Martin JML Handy NC The role of the basis set: Assessing density functional theory J. Chem. Phys 2003 119 3005 3015

[55] Tschumper GS Schaefer HF III Predicting electron affinities with density functional theory: Some positive results for negative ions J. Chem. Phys 1997 107 2529 2541

[56] Pak C Xie Y Van Huis TJ Schaefer HF Electron affinities of the bromine fluorides, BrF_n_ (n = 1–7) J. Am. chem. Soc 1998 120 11115 11121

[57] Roos BJ Ab Initio Methods in Quantum Chemistry Lawley KP John Wiley & Sons New York, USA 1987 399 445

[58] Guha S Francisco JS Structures, vibrational spectra, and relative energetics of CH_3_BrO_3_ isomers J. Phys. Chem A 2000 104 3239 3245

[59] Chase MW Davies CA Downey JR Frurip DJ McDonald RA Syverud AN NIST-JANAF Thermochemical Tables J. Phys. Chem. Ref. Data 1985 1 146 147

[60] Ruscic R Berkowitz J Experiment determination of ΔH_f_θ (HOBr) and ionization potentials (HOBr): Implications for the corresponding properties of HOI J. Chem. Phys 1994 101 7795 7803

[61] Chase MW Davies CA Downey JR Frurip DJ McDonald RA Syverud AN JANAF Thermochemical Tables, 3rd ed J Phys Chem Ref Data 1985 14 Suppl. 1 8 9

